# Heterosis as a consequence of regulatory incompatibility

**DOI:** 10.1186/s12915-017-0373-7

**Published:** 2017-05-11

**Authors:** Rebecca H. Herbst, Dana Bar-Zvi, Sharon Reikhav, Ilya Soifer, Michal Breker, Ghil Jona, Eyal Shimoni, Maya Schuldiner, Avraham A. Levy, Naama Barkai

**Affiliations:** 1 Department of Molecular Genetics, Weizmann Institute of Science, Rehovot, 7610001 Israel; 2 Plant and Environmental Sciences Department, Weizmann Institute of Science, Rehovot, 7610001 Israel; 3 Broad Institute of MIT and Harvard, Cambridge, MA 02142 USA; 4 Department of Systems Biology, Harvard Medical School, Boston, MA 02114 USA; 5 Department of Life Sciences Core Facilities, Weizmann Institute of Science, Rehovot, 7610001 Israel; 6 Department of Chemical Research Support, Weizmann Institute of Science, Rehovot, 7610001 Israel; 7 Current affiliation: Calico Labs, South San Francisco, CA 94080 USA

**Keywords:** Hybrid vigor, Heterosis, Incompatibility, Budding yeast

## Abstract

**Background:**

The merging of genomes in inter-specific hybrids can result in novel phenotypes, including increased growth rate and biomass yield, a phenomenon known as heterosis. Heterosis is typically viewed as the opposite of hybrid incompatibility. In this view, the superior performance of the hybrid is attributed to heterozygote combinations that compensate for deleterious mutations accumulating in each individual genome, or lead to new, over-dominating interactions with improved performance. Still, only fragmented knowledge is available on genes and processes contributing to heterosis.

**Results:**

We describe a budding yeast hybrid that grows faster than both its parents under different environments. Phenotypically, the hybrid progresses more rapidly through cell cycle checkpoints, relieves the repression of respiration in fast growing conditions, does not slow down its growth when presented with ethanol stress, and shows increased signs of DNA damage. A systematic genetic screen identified hundreds of *S. cerevisiae* alleles whose deletion reduced growth of the hybrid. These growth-affecting alleles were condition-dependent, and differed greatly from alleles that reduced the growth of the *S. cerevisiae* parent.

**Conclusions:**

Our results define a budding yeast hybrid that is perturbed in multiple regulatory processes but still shows a clear growth heterosis. We propose that heterosis results from incompatibilities that perturb regulatory mechanisms, which evolved to protect cells against damage or prepare them for future challenges by limiting cell growth.

**Electronic supplementary material:**

The online version of this article (doi:10.1186/s12915-017-0373-7) contains supplementary material, which is available to authorized users.

## Background

Hybrids between related species or strains often display traits that are superior to their parents, in particular in relation to growth vigor. This phenomenon, known as heterosis, has been observed in all eukaryotic kingdoms, namely plants, animals, and fungi. Hybrids and heterosis have fascinated evolutionary biologists since Darwin [1, 2] and continue to fascinate modern day geneticists. Moreover, hybrid vigor has been extensively exploited for increasing productivity in agriculture [3, 4]. Therefore, identifying the underlying mechanism of hybrid vigor is of great interest [5].

Hybridization plays an important role in the emergence of new species by providing a myriad of novel interactions giving rise to new phenotypes, thereby helping to colonize unoccupied ecological niches [6–8]. Hybrid vigor can give an advantage to the hybrids in certain niches, and hybrid incompatibilities can secure their reproductive isolation [8–12].

Heterosis is often viewed as the opposite of hybrid incompatibility, the more expected clash between genomes [5]. The common view, “the dominance model”, describes heterosis as the opposite of inbreeding depression, i.e., in the hybrid state, deleterious mutations specific to one parent are complemented by the wildtype dominant allele of the other parent [13, 14]. A second class of models [5] attributes heterosis to overdominance or epistatic effects, in which interactions between alleles of the two parents, either coding for the same gene or for different genes, emerge in the hybrid and lead to its superior performance. Studies of heterosis in different plant species provide support for both the dominance and overdominance model by defining specific alleles that contribute to heterosis [15–17]. Still, these models fail to explain phenomena such as the maintenance of heterosis after deleterious mutations are purged or progressive heterosis in polyploids [5].

To gain a broader view on how gene regulation is altered following hybridization, previous studies have applied genome-wide profiling approaches. These studies revealed large-scale differences between hybrids and their parents in gene expression [18, 19], nucleosome positioning [20], and other genomic features [21, 22]. The unique expression pattern in hybrids was attributed mainly to differences between the parents, namely that most genes were expressed at a level that was intermediate between the two parents. An additional fraction of genes showed a distinct expression, being expressed more or less than both parents, likely resulting from novel cis-trans interactions that emerged in the hybrid due to the mixing of the two parental genomes. Gene expression rewiring could affect regulatory mechanisms and thereby contribute to the emergence of novel phenotypes, including heterosis. The contribution of these expression changes to growth performance is not well understood.

The widespread differences in gene expression between the hybrid and its parents raised the question of how heterotic loci are distributed across the genome. Can we attribute heterosis to a small number of genes or is it the result of multiple effects distributed across many alleles? Are heterotic effects confined to specific functional groups, and if so, which? To examine this, we turned to budding yeast, where systematic genetic screens are more easily performed. Heterosis is relatively common in budding yeast, in particular in hybrids between domesticated strains [23, 24]. In fact, a recent study observed growth in 35% of the 120 intra-specific crosses between different strains analyzed [25]. Inter-specific hybrids are also widespread in domesticated strains used for the making of alcoholic beverages, bread, and biofuel [26], but they have received less attention in heterosis research.

Budding yeast hybrids are easily generated in the laboratory, and in our initial studies we observed a particular hybrid between *Saccharomyces cerevisiae* and *Saccharomyces paradoxus* that grew faster than both its (fast growing) diploid parents under multiple conditions. Using this model, we systematically examined the contribution of all non-essential *S. cerevisiae* alleles to hybrid growth. This was done by generating a hemizygote hybrid library, in which each individual hybrid was lacking a specific *S. cerevisiae* allele, yet included the corresponding *S. paradoxus* one. Generation of this library was made possible by systematically mating a wildtype *S. paradoxus* haploid strain with the deletion library available in *S. cerevisiae*, which enabled the creation of a hemizygote library of hybrids. Using competition assays, we identified hemizygote deletions that impair the growth specifically in the hybrid background, but had no effect when lacking one copy in the diploid *S. cerevisiae* background. Hundreds of such alleles that specifically contributed to the growth in the hybrid background, defined here as “heterotic alleles”, were identified. Notably, these heterotic alleles largely differed between growth conditions. The multiplicity of allele effects that were specific to the hybrid, together with their functional associations, suggest that the hybrid not only experiences growth heterosis but also incompatibilities that perturb regulatory mechanisms, a notion that is supported by our phenotypic analysis. Based on these results, we propose that hybrid-incompatibility directly contributes to heterosis; specifically, heterosis would result from incompatibilities that target processes that protect cells against damage or prepare for future challenges, which often come at the expense of rapid growth. Perturbation of these mechanisms in the hybrid background would therefore appear superior, as they increase growth, but would come at the expense of limiting the protecting mechanism.

## Results

### A budding yeast hybrid showing growth heterosis

We generated hybrids by mating haploids of *S. cerevisiae* and *S. paradoxus*, two closely-related *sensu-stricto* species that express largely the same set of genes, with 90% and 80% sequence identity in coding or inter-genic regions, respectively [27]. The hybrids are limited in meiosis, producing less than 1% viable gametes [28], but can propagate vegetatively without signs of genetic instability or aneuploidy.

**Fig. 1:**
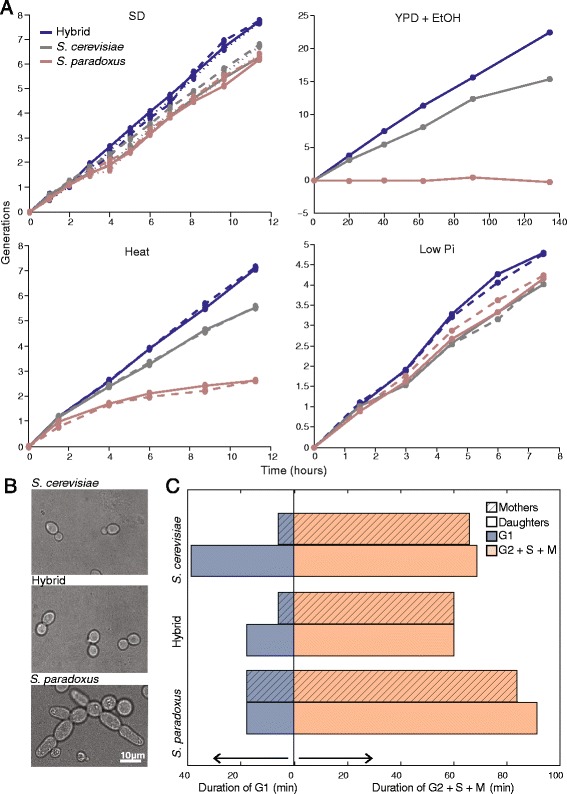
Growth heterosis in a yeast hybrid. **a** Growth heterosis in the yeast hybrid in rich and stress media. Cells were diluted into rich (SD, YPD) or stress media (YPD + 7.5% ethanol, YPD in 37 °C, 0.2 mM Pi), and doubling time was measured by following their optical density (OD). Cultures were diluted periodically to maintain the cells in logarithmic phase for the duration of the experiment. Different repeats indicate biological replicas. **b** Growth pattern of the hybrid and its diploid parents presented with rich (SD) media. **c** Perturbed cell-cycle delays in hybrid. Shown are the median cell-cycle times of the unbudded (G1) and budded (S + G2 + M) hybrid and its diploid parents. Mother and daughters are shown separately. Data extracted from live-cell microscopy (see Additional files 2, 3 and 4: Movies S1–S3, and time distributions in Additional file 1: Figure S1B)



When provided complete media (SD), both diploid parents grow with a division time of approximately 90 minutes, typical of rapidly growing strains. Still, the hybrid grows approximately 20% faster than both diploid parents (one-way ANOVA, *P* < 10^–7^) (Fig. 1a and Additional file 1: Figure S1A). Growth heterosis was observed also in other conditions, including high temperature, high ethanol concentrations, and low Pi concentrations (Fig. 1a). We used live-cell microscopy to quantify the duration of the different cell cycle phases (Fig. 1b, Additional files 2, 3 and 4: Movies S1–S3). The two parents regulate their cell cycle differently; *S. cerevisiae* cells have short G2 phase, thereby generating small daughter cells that extend their G1 phase to retrieve their mother’s size. *S. paradoxus* cells, on the other hand, regulate their size by extending the G2 phase, followed by a short G1 [29]. We find that the hybrid G2 phase was as short as in *S. cerevisiae*, yet it did not extend its daughter’s G1, which was as short as in *S. paradoxus* (Fig. 1c and Additional file 1: Figure S1B).

**Additional file 2: Movie S1.** Growth of *S. paradoxus*. Note the pseudo hyphae-like growth. (M4V 2559 kb)

**Additional file 3: Movie S2.** Growth of *S. cerevisiae*. Note *S. cerevisiae*’s asymmetrical division. (M4V 766 kb)

**Additional file 4: Movie S3.** Growth of the hybrid. Note the synchronized growth in the hybrid due to loss of G1 and G2 delay in the daughter cells. (M4V 455 kb)


**Fig. 2:**
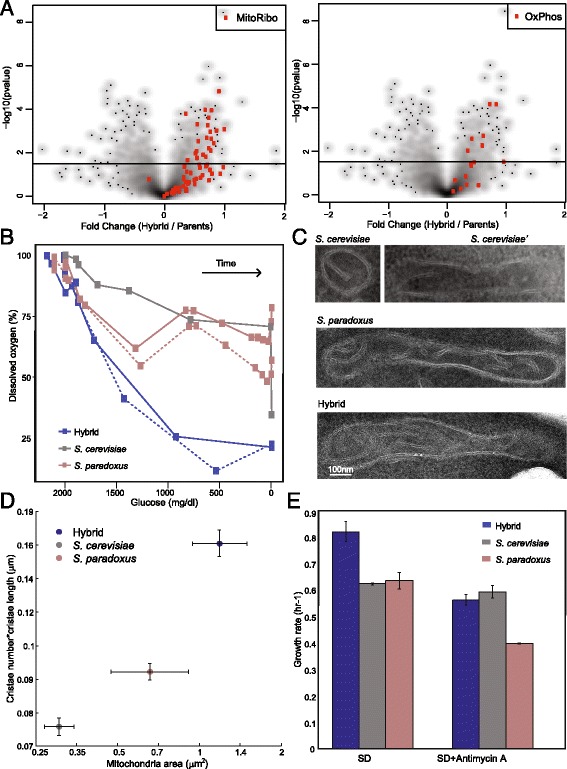
Perturbed glucose-dependent respiration repression in the hybrid. **a** Reduced glucose-repression through upregulation of respiration gene expression in the hybrid. Each point represents a gene from the indicated group. Fold expression changes were calculated between the hybrid and the mean expression of *S. cerevisiae* and *S. paradoxus*. See Additional file 5: Table S1 for gene names and expression values. Data from [18]. **b** The hybrid consumes oxygen in the presence of glucose, but not its diploid parents. Shown are measurements of glucose and dissolved oxygen for cultures incubated into glucose-containing rich media (SD). Measurements were made during batch growth within a fermenter. **c**, **d** Hybrid mitochondria are larger and contain more cristae. **c** Representative electron microscopy (EM) images of mitochondria in the hybrid and its diploid parents grown in SD. **d** Quantification of mitochondria area and cristae of the hybrid and the diploid parents, from EM images. (N = ~20 mitochondria per strain). **e** Heterosis is lost when respiration is inhibited. Growth rates are shown for cells growing in SD in the presence or absence of the respiration inhibitor Antimycin A (N = 3)

Sustained rapid growth entails a more efficient production of biomass. Biomass and energy production are regulated by the routing of carbon through central carbon metabolism. We previously noted that respiratory gene expression is higher in the hybrid relative to its parents, even when grown in glucose (Figure S13 in [18], Fig. 2a), which was surprising, as budding yeast exhibits glucose-mediated catabolite repression of the TCA cycle. We therefore asked whether glucose repression is reduced in the hybrid, enabling more efficient energy generation through respiration. Measuring oxygen usage along the growth curve confirmed that hybrids consumed oxygen at a high rate throughout the growth curve, even when glucose was abundant, in contrast to *S. cerevisiae* and *S. paradoxus*, where oxygen consumption was lower when glucose was present (Fig. 2b and Additional file 6: Figure S2A, B). Consistently, hybrid mitochondria were larger and contained more cristae compared to *S. cerevisiae* and *S. paradoxus*, as visualized by electron microscopy (Fig. 2c, d and Additional file 6: Figure S2C). Finally, heterosis was reduced upon the addition of the respiration blocker Antimycin A to the level of the best parent (Fig. 2e and Additional file 6: Figure S2D). Together, our results suggest that reduced glucose repression in the hybrid enables it to respire even in the presence of glucose.

**Fig. 3:**
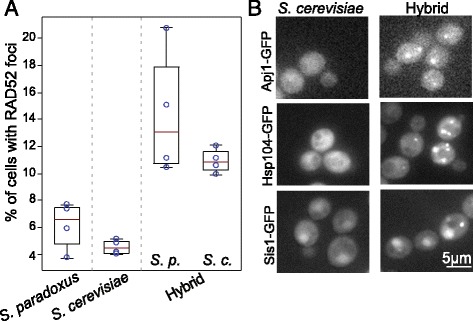
Increased DNA damage in the hybrid. **a** Increased formation of foci indicative of DNA damage in hybrid. Fraction of cells showing *RAD52-GFP* foci in the hybrids and its diploid parents grown in SD (N = 4, ~2000 cells). For the hybrid, the results are shown when tagging either the *S. cerevisiae* or *S. paradoxus* allele. **b** The hybrid shows signs of DNA damage stress through protein localization. Chaperones that are known to localize into punctates during DNA damage are observed in the hybrid (Additional file 6: Table S2)

Increased respiration may lead to oxidative stress that can cause strand breaks in DNA. Therefore, we assessed DNA damage in the hybrid by following Rad52-GFP, a protein that localizes to foci of DNA double-strand breaks [30]. Notably, the frequency of cells with Rad52-GFP foci increased by approximately two-fold in the hybrid compared to either parent (Fig. 3a). Further, we noted that, in the hybrid, a group of cytosolic chaperones were localized into punctate structures, a known marker for stress, such as DNA damage (Fig. 3b and Additional file 7: Table S2).

### A genome-wide genetic screen detects hundreds of alleles contributing specifically to hybrid growth in an environment-dependent manner

Classical models of heterosis, including dominance, overdominance, or dosage models, attribute the hybrid’s superior performance to the action of specific alleles. We reasoned that, working with budding yeast, we could systematically screen for heterotic alleles, defined here as alleles that contribute to the hybrid’s growth but do not show a dosage effect in the parental background. This general definition includes alleles that function through dominance, dosage, overdominance, epistasis, or more complicated (e.g., cis and trans) effects.

**Fig. 4:**
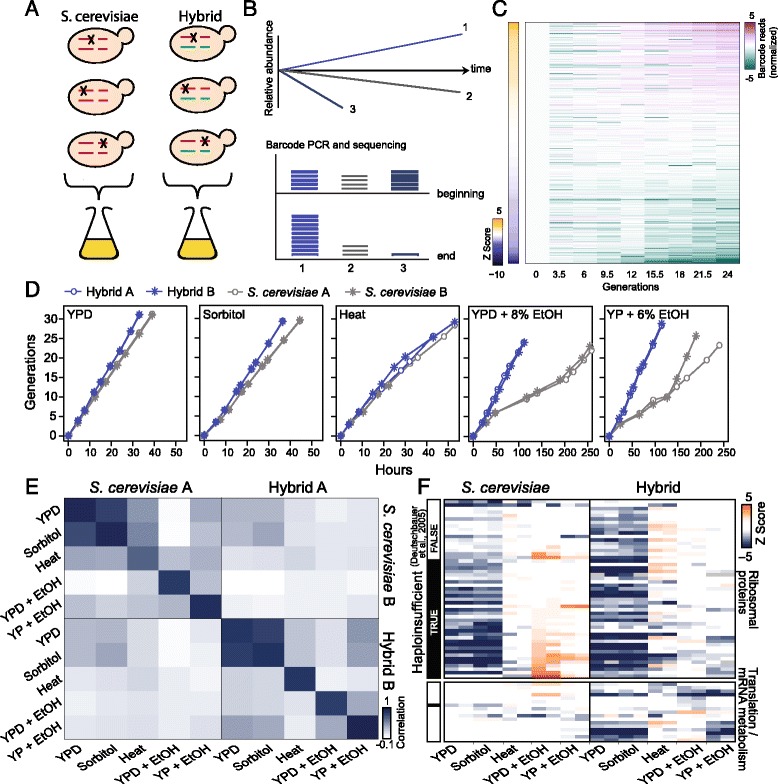
Genome-wide screen for alleles contributing to hybrid growth. **a**, **b** Screen for *S. cerevisiae* alleles contributing to hybrid growth. A hemizygote hybrid library was constructed by mating *S. paradoxus* with a library of *S. cerevisiae* strains, each of which was a deletion of one *S. cerevisiae* gene. Each strain was labeled by a specific barcode, enabling the measurements of relative strain abundances within a co-growing pool using sequencing. **c** Change in abundance of individual hemizygote strains. A pool of hybrid hemizygotes was grown in YPD + 8% ethanol. Shown are the (log) normalized barcode reads of 100 strains showing the most pronounced increase or decrease in frequency, together with 200 control strains that maintained their abundance throughout the experiment. The *Z*-score of the calculated growth rate for each strain is shown in the sidebar. **d** Growth curves of hemizygote hybrid and *S. cerevisiae* pools. The two hemizygote pools were kept for approximately 30 generations in the logarithmic phase through subsequent dilution, and sampled every three generations. Two independent biological replicates are shown. **e** Correlation between growth rates of hemizygote strains. Note the reproducibility between the two independent repeats, contrasting the low-correlation when comparing different conditions or backgrounds. **f** Hybrid-specific dosage sensitivity. Shown are z-scores of growth rates of strains hemizygous for genes coding for ribosomal proteins and mRNA metabolism, as indicated, that show reproducible effects in at least one condition (*Z* Score < –1.5; see Additional file 11: Table S5 for gene list). Missing values are shown in black. Sidebar marks previously defined haploinsufficient genes [34]

Our screen is based on the availability of a deletion library, corresponding to all non-essential *S. cerevisiae* genes. Starting from this library, we generated a library of hemizygote hybrids that lack a specific *S. cerevisiae* allele but contain the corresponding *S. paradoxus* allele, and a control library containing hemizygote *S. cerevisiae* diploids (Fig. 4a). Two independent libraries were generated for each genetic background.

The deletion library was specifically designed to enable sensitive measurement of the growth rate of individual strains, while growing all strains in one pool [31, 32]. This approach has the advantage that all strains are exposed to the same environment, making a comparison between the individual strains more controlled. Specifically, each strain in the library is marked by a specific sequence barcode that is flanked by a common sequence, so that high-throughput sequencing can be used to quantify the relative abundance of each strain within the growing pool (Fig. 4b, c, see Methods and Ref [33]). Temporal changes in a strain’s abundance during pool growth indicate its growth rate relative to the pool’s average, i.e., slow growing strains will be gradually outcompeted, while the fast growing ones will become increasingly more abundant. Note that the measured growth rate of the pool will approximate the wild-type growth rate as the majority of mutants do not show a growth defect at any given condition [33].

We tested strain performance under five growth conditions (YPD, YPD + Sorbitol 1 M, YPD at 37 °C, YPD + 8% ethanol, and YPD + 6% ethanol; Fig. 4d, Additional files 8 and 9: Table S3 and Table S4). In each condition, the pools were maintained in log-phase throughout the experiment by back-dilution (see Methods) to ensure constant conditions and limit possible effects of nutrient depletion or secretion. In total, 865 alleles reduced growth in at least one condition or genetic background (*Z* Score < –1.5 in both biological replicates, Additional file 10: Figure S3A). These genes were classified into functional groups based on databases and literature (Additional files 10 and 11: Figure S3B and Table S5). Inferred growth rates were highly correlated between two replicates corresponding to two independently pooled libraries.

The effects that the different alleles had on growth differed between the conditions (Fig. 4e). For example, stress conditions, such as heat and high ethanol concentration, increased dosage sensitivity to genes involved in peroxisome function, cell wall formation/breakdown, or protein and lipid modifications (Additional file 10: Figure S3C). Most notably, while previously annotated haploinsufficient genes in *S. cerevisiae* [34], such as ribosome components, were reproducibly identified as dosage sensitive in rich media, these strains showed no effect in growth conditions in which cells were growing more slowly (Fig. 4f and Additional file 10: Figure S3D). Thus, depending on the growth conditions, loss of one allele could range from deleterious to even being beneficial.

Surprisingly, perhaps, the set of genes sensitive to hemizygosity in the hybrid greatly differed from that of its *S. cerevisiae* parent, even within the same growth condition (Fig. 4e, f). Thus, hundreds of *S. cerevisiae* alleles contribute to hybrid growth but not to *S. cerevisiae* growth. These alleles are not confined to a single pathway, but are associated in multiple cellular processes (Fig. 4f and Additional file 10: Figure S3B, C).

### Heterotic alleles consistent with hybrid phenotypes

**Fig. 5:**
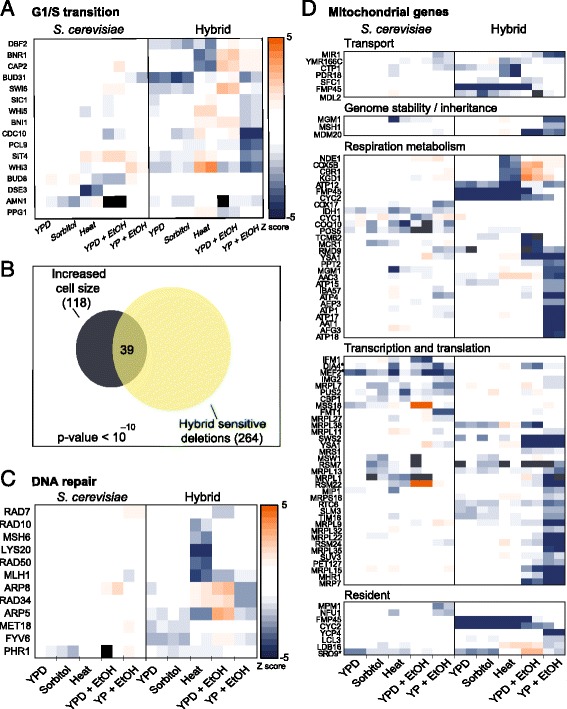
Gene sensitivity results indicate perturbation in several pathways. **a** The hybrid shows increased sensitivity to cell-cycle genes. Shown are z-scores of growth rates of strains hemizygote for genes coding for the G1/S transition that show reproducible effects in at least one condition. Missing values are shown in black. **b** The hybrid shows increased sensitivity to genes increasing cell size. Genes reported to increase cell size [50] are highly enriched within the set of genes showing hybrid-specific dosage sensitivity in at least one of the conditions (hypergeometric test). This increased sensitivity is independent of the functional association of these genes. **c** The hybrid shows high sensitivity to DNA damage genes, same as Fig. 5a for the indicated strains. **d** Hybrid hemizygote strains show high sensitivity to mitochondrial genes, same as Fig. 5a for the indicated genes, which code for proteins that localize to the mitochondria and are either nuclear or mitochondrial encoded

We observed a correspondence between allele-specific sensitivity and the hybrid phenotypes noted above. First, the hybrid shows increased sensitivity to alleles involved in the G1/S transition (Fig. 5a) and to alleles whose deletion increases cell size, irrespectively of their functional association (hypergeometric *P* value: Hybrid < 10^–10^, *S. cerevisiae*: 0.65, Fig. 5b). This increased sensitivity may be explained by the shorter duration of this cell cycle phase in the hybrid compared to *S. cerevisiae*, i.e., as the G1/S transition is a major checkpoint where nutritional status and cell-size are monitored and its duration is correlated to birth size [35, 36], the hybrid’s shorter G1/S duration may limit the checkpoint capacity to correct for size perturbations that would require shortening its duration beyond the functional limit.

Also connected to cell cycle progression, the hybrid showed an increased sensitivity to DNA repair genes (Fig. 5c). This, together with the phenotypic results of increased presence of DNA damage markers in the hybrid (Fig. 3a, b), may suggest suboptimal performance of mechanisms that maintain genome integrity.

Finally, consistent with the reduced glucose repression seen in the hybrid, the hybrid was sensitive to the deletion of *S. cerevisiae* alleles coding for genes that are localized to mitochondria. This sensitivity exceeds that of its *S. cerevisiae* parent (*P* value < 10^–2^), and was particularly pronounced during growth on ethanol, but observed also on glucose (Fig. 5d).

### The hybrid escapes a programmed cell cycle slow-down under severe ethanol stress

**Fig. 6:**
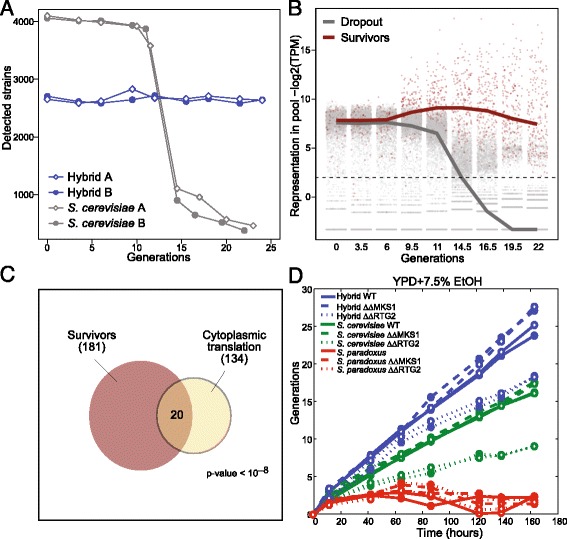
Hybrid does not slow-down its growth during ethanol stress. **a**, **b** A minority of hemizygote strains overtakes the *S. cerevisiae* pool when subject to ethanol stress. **a** Number of strains that were reliably identified (>30 reads) when sequencing the hemizygote hybrid or *S. cerevisiae* pools at different time points in YPD + 8% ethanol medium. **b** Relative representation of each strain within the *S. cerevisiae* pool. Red points represent the strains that were abundant (log_2_(TPM + 1) > 2) at the last two time points in both *S. cerevisiae* experiments. **c** Strains deleted for genes coding for ribosomal proteins are enriched in the survivor pool. Shown is the hypergeometric *P* value for enrichment of ribosomal genes. **d** Increasing retrograde signaling improves hybrid growth under ethanol stress. Cultures were diluted periodically to maintain the cells in logarithmic growth. MKS1 is a negative regulator, whereas RTG2 is an activator of retrograde pathway. ΔΔ refers to strains deleted in both alleles of the gene

The largest difference in the pattern of allelic sensitivity between the hybrid and *S. cerevisiae* was observed under conditions of high ethanol stress. Under this condition, a reproducible minority of hemizygote strains overtook the population in the two *S. cerevisiae* replicates (Fig. 6a, b and Additional files 12 and 13: Figure S4A, B, Table S6). In contrast, the hybrid showed the typical pattern of allele sensitivity as seen in other conditions. This differential pattern of effects was also reflected in the overall growth of the pools (cf. Figs. 1a and 4d); while the hemizygote hybrids maintained steady growth throughout the experiment, similarly to all other conditions tested, growth of the hemizygote *S. cerevisiae* pool was initially rapid, then slowed down, and became rapid again after approximately 10 generations. The rapid growth of the hybrid in high ethanol is especially striking considering that the *S. paradoxus* parent fails to grow in this condition (Fig. 1a). Note that, as during most of the growth phase cells were maintained at low density, the possible metabolism of ethanol by the cells had no effect on ethanol concentration.

The hybrid therefore maintains a stable growth in high ethanol concentration, while the *S. cerevisiae* diploids slowdown their growth after some period. Notably, this slowdown can be overcome by decreased expression of individual genes. This unique dosage response suggests that the growth slow-down is an active and adapted strategy and not a passive reaction to unavoidable toxicity. In support of that, strains that are maladapted in rich conditions were enriched amongst the surviving hemizygote diploids (Figs. 4f and 6c), which may also explain the selection of euploid *S. cerevisiae* x *S. uvarum* hybrids upon ethanol stress [37].

To try and identify the basis of this increased ethanol resistance in the hybrid, we examined the pattern of allelic sensitivity. The hybrid showed an increased dependency on retrograde signaling (Additional file 12: Figure S4C). This response is triggered by damaged mitochondria to induce nuclear-encoded protecting mechanisms [38]. Induction of this pathway within the hybrid could render the hybrid more stress resistant. In support of that, over-activating the retrograde pathway increased growth under ethanol stress for both the hybrid and its *S. cerevisiae* diploid parent, although accounting for only a fraction of hybrid growth vigor (Fig. 6d and Additional file 12: Figure S4D).

## Discussion

### The genetic control of heterosis

We describe a systematic analysis of hybrid growth in the absence of a specific allele from the *S. cerevisiae* parent. This systematic screen was enabled by the availability of a gene deletion library in *S. cerevisiae*, previously generated by a large community effort. Using this library, we generated a hemizygote library of hybrids, each of which lacked a specific *S. cerevisiae* allele, and tested the growth rate of each stain under competitive conditions. Although we did not have the complementary library of hemizygotes lacking the *S. paradoxus* allele, our analysis provided us with a comprehensive characterization of all *S. cerevisiae* alleles that contribute to hybrid growth. Notably, this screen enables testing of the effect of a genetic locus in the relevant hybrid background rather than in segregating populations or after introgression in a non-hybrid background (e.g., recombinant inbred or introgression lines).

We initially expected that dosage sensitivity would be largely similar between the *S. cerevisiae* parental and hybrid background, allowing us to identify a limited number of alleles that contribute to heterosis through dominance or partial dominance effects. In striking contrast to these expectations, we observed that hundreds of loci were sensitive to hemizygosity in the hybrid but not in its *S. cerevisiae* parent, even within the same growth condition (Fig. 4e, f). Although we cannot exclude that this difference could be explained by differential dosage effects in the *S. paradoxus* parent, which we could not test, we find this unlikely, in particular when considering the fact that the set of hemizygote-sensitive genes in both the hybrid and *S. cerevisiae* parent reproducibly varied between conditions.

One interpretation of our data is therefore that dominance effects are abundant in this hybrid, as has been proposed previously for yeast hybrids [23, 24]. Several reasons, however, argue against this interpretation. First, the number of alleles that had an effect in the hybrid but not in the *S. cerevisiae* parent was equivalent to the number of alleles that affected the *S. cerevisiae* parent but not the hybrid. Second, the differences in allele effects between the hybrid and its *S. cerevisiae* parent were similar to the differences in allele effects between *S. cerevisiae* cells growing in different conditions. Finally, both parents grow at a rate that is characteristic of fast growing strains, suggesting that they do not contain a large number of deleterious alleles. We therefore favor the alternative possibility that most effects represent differential dosage sensitivity between the hybrid and its *S. cerevisiae* parent. Recent studies on the *SINGLE FLOWER TRUSS* locus in tomato also support the importance of dosage optimality in heterosis [39, 40].

### Perturbation of regulatory mechanisms in the hybrid

Regardless of the mode of action, the abundance of alleles that contribute to hybrid growth and their distribution across the genome suggests that the mixing of the two parental genomes impacts multiple cellular processes. This conclusion is consistent with a recent study in rice showing a large number of loci involved in heterosis, whose identity differs among different crosses [16]. A common theme emerging from our genetic and phenotypic data is that key cellular functions are perturbed in the hybrid, including (1) repression of respiration being largely alleviated, allowing cells to respire even when glucose is present; (2) perturbation of cell size-dependent timing of the cell cycle G1/S phase; and (3) continuation of growth under ethanol stress, unlike the parental species, which slows down.

These perturbations of key regulatory programs identified, together with the apparent large-scale rewiring of allelic (or dosage) effects, make the heterotic phenotype even more surprising. Indeed, naively, if the parents are evolutionarily optimized for rapid growth, such wide-ranging perturbations would be expected to reduce, rather than increase, the growth rate. A possible explanation is the role of tradeoff in defining cellular growth. A general scheme that emerges from recent studies is that cells often compensate their fast growth in order to protect against damage, or to prepare for possible future challenges. If hybrid incompatibility perturbs the function of these regulatory processes, a faster growth will ensue. Regulatory mechanisms that limit biomass production or growth are likely to diverge more readily and therefore be more amendable to perturbations in the hybrid.

This appears consistent with the phenotypes observed, namely that limiting respiration, when oxygen is available, may act as a mechanism to competitively interfere with other microbes using a resource that itself later becomes accessible as a growth substrate. Prolonged G1 (or G2) increases division time, but allows regulation of the cell size, such as correcting for large fluctuations in size. Slowing down the cell cycle during ethanol stress may prevent damage, perhaps explaining why it was not reverted during evolution.

In the case of *S. cerevisiae* and *S. paradoxus* we hypothesize that heterosis arises from adaptive trade-offs. Specifically, heterosis may be a reflection of the different tradeoffs that govern evolution. While growth rate needs to be maximized, this maximization is subject to some constraints, such as maintaining genome stability, a process that may indeed be limited in the hybrid, as we showed. Consistent with this hypothesis, studies of plant hybrids also show a tradeoff between growth and stress response [41]*.* Furthermore, a role of evolutionary tradeoffs in constraining agriculture-relevant traits, such as biomass growth, is well appreciated from studies of crop and animal breeding. Selecting for plants for maximal agriculture productivity, for example, reduces their fitness under natural conditions [42, 43].

**Fig. 7:**
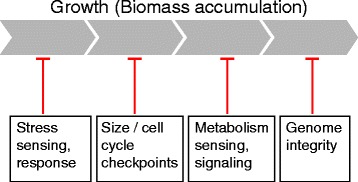
Model of heterosis – hybrid incompatibilities perturbing failsafe mechanisms limit growth of the wild-type background

Taken together, our study suggests that heterosis and incompatibility may be tightly linked, namely that incompatibilities that perturb regulatory mechanisms may contribute to hybrid growth vigor (Fig. 7).

## Conclusions

Our results define a budding yeast hybrid that is perturbed in multiple regulatory processes but still shows a clear growth heterosis. Our study suggests that incompatibilities that perturb regulatory mechanisms may contribute to hybrid growth vigor. An incompatibility-based explanation of heterosis may account for results that were not predicted from the dominance/complementation model such as the maintenance of heterosis after deleterious mutations are purged or progressive heterosis in polyploids [5].

## Methods

### Generation of hybrids

Hybrids were generated by mating haploid *S. cerevisiae* 4741 HO::Kan^r^ with haploid *S. paradoxus* CBS432 Matα HO::Nat^r^. The two haploid parent strains, each containing a different antibiotic resistance, were mixed on a YPD agar plate and grown for 7 hours at 30 °C. The yeasts were then transferred to YPD agar plates with double selection (YPD + 0.1 mg/mL Nourseothricin (Nat, WERNER BioAgents) + 0.2 mg/mL G418 (Calbiochem)) to select for hybrid progeny.

### Growth curves

The hybrid and the diploid parents were grown overnight in YPD, back-diluted to OD_600_ 0.05 in YPD, and grown for 5 hours at 30 °C, in order to allow the cultures to reach log phase. Cells were then diluted in the appropriate media for growth in stress media. Cells were washed twice in water previous to dilution. Cultures were grown at 30 °C unless otherwise indicated. The cultures were kept in log phase by back-diluting to OD_600_ = 0.05 when a culture reached OD_600_ ~ 1.

### Cell cycle analysis using high throughput time-lapse microscopy

Olympus IX71 microscope was automated using a motorized XY stage (Prior), fast laser autofocus attachment [44], excitation and emission filter wheels (Prior), and shutters (UniBliz). The EMCCD camera was an AndoriXon with a pixel size of 16 and 512 × 512 EMCCD chip cooled to –68 °C. eGFP and mCherry were detected using EXFO X-Cite 120 light source at 12.5% intensity using Chroma 89021 mCherry/GFP ET filter set. Exposure time for the detection of eGFP and mCherry was 100 msec. The cells were observed using a 60 × 0.9 NA UPLFLN/APO objective. The microscope was controlled by custom written software running on Red Hat Linux. The fast autofocus and filter switching times allowed simultaneous imaging of 60 fields of view with a time resolution of 3 minutes.

Preparation of cells for time lapse imaging was performed as previously described [45]. Briefly, log phase cultures were seeded at OD_600_ ~ 1 on a slab of 2% low melt agarose containing SC and imaged between the agar pad and the cover glass. Bright field images were taken 1 μm below the focal plane to facilitate image analysis. This time-lapse setup allows unperturbed exponential growth in a single plane throughout most of the experiment. Image analysis for the hybrid and *S. cerevisiae* was performed as previously described [36], whereas image analysis for *S. paradoxus* was performed by manually tracking the cell division.

### Respiration inhibition using Antimycin A

Three biological replicates of each strain were grown overnight in SD and back-diluted to OD_600_ = 0.05 in 20 mL YPD. After allowing the cultures to reach log phase, they were washed in water and diluted to OD_600_ = 0.01 in SD + 10 μM Antimycin A (Sigma) media. The cultures were kept in log phase by back-diluting to OD_600_ = 0.01 when a culture reached OD_600_ ~ 1. OD_600_ measurements were taken every 90 minutes.

### Counting *RAD52* foci as indication of double strand breaks

Strains 4741 *S. cerevisiae* RAD52-YFP and CBS432 OS142 *S. paradoxus* Mata RAD52-YFP were constructed. Each of the strains was mated with Matα *S. paradoxus* and *S. cerevisiae.* In order to select diploid progenies following the mating, single colonies were collected and their DNA was stained in order to differentiate between diploid and haploid colonies using flow cytometry. Once in log phase, cells were incubated in 70% ethanol for 1 hour at 4 °C, incubated in RNase A 1 mg/mL for 40 minutes at 37 °C, incubated in Proteinase K 20 mg/mL for 1 hour at 37 °C, and followed by 1 hour incubation in SYBR green (S9430, Sigma-Aldrich) (1:1000) at room temperature in the dark. Cells were washed twice with 50 mM Tris-HCl pH 8 between each incubation. The stained cells were sonicated in Diagenode Bioruptor for three cycles of 10” on and 20” off at low intensity. The fluorescence was measured using BD LSRII Flow Cytometer (BD Biosciences). After diploid colonies were picked, cells were grown in SD to log phase and were imaged in OD_600_ = 0.150. Images were acquired using Olympus IX83 based Live-Imaging system equipped with CSU-W1 spinning disc (sCMOS digitale Scientific Grade Camera 4.2 MPixel VS LaserModule 1863C with LaserMerge System Laser module, laser 488 nm with 100 mW). The number of cells containing *RAD52* foci was counted.

### Mitochondrial morphology using electron microscopy

Cells were grown in SD medium. Once they reached OD_600_ ~ 0.5, 5 mL of a fixative solution (6% paraformaldehyde, 4% glutaraldehyde and Cacodylate buffer 0.2 M (pH = 7.4)) was added to 5 mL of media. The samples were gently rotated for 40 minutes at 30 °C, then centrifuged and washed twice in Cacodylate buffer and incubated again for an hour in the fixative solution. After washing with Cacodylate buffer and centrifugation, samples were embedded in 10% gelatin in water, fixed overnight with the fixative solution, washed in Cacodylate buffer, and then incubated overnight in 2.3 M sucrose and rapidly frozen in liquid nitrogen. Frozen ultrathin (70–90 nm) sections were cut with a diamond knife (Diatome AG, Biel, Switzerland) at –120 °C on an EM UC6 ultramicrotome (Leica Microsystems, Vienna, Austria). The sections were collected on 200-mesh Formvar-coated nickel grids. Contrasting and embedding were performed as previously described [46]. The embedded sections were observed in a Tecnai T12 electron microscope (FEI, Eindhoven, The Netherlands) operating at 120 kV. Images were recorded using an Erlangshen ES500W CCD camera (GATAN) or an Eagle 2 k × 2 k CCD camera (FEI). The quantification of mitochondrial area and cristae was performed using imageJ [47].

### Cell cycle imaging for time-lapse movie and stills

Cells were grown in SD to reach log phase. Samples with OD_600_ ~ 0.02 were seeded on a slab of SD + 2% slab low melt agarose. Images were taken in an Olympus IX83 based Live-Imaging system equipped with CSU-W1 spinning disc: sCMOS digital Scientific Grade Camera 4.2 MPixel. The cells were kept at 30 °C using in-stage incubator Chamlide TC. Images were taken every 5 minutes. Movie cropping and labeling was performed using imageJ [47].

### Measuring oxygen and glucose consumption

The fermentation experiments were performed using a DASBox mini Bacterial Fermentation system (DASGIP, Eppendorf), with the online monitoring and control of temperature, dissolved oxygen (DO), OD, aeration, and mixing. Yeast cells were grown in the fermenters in 200 mL SC media supplemented with 2% glucose, using the following controlled parameters: 30 °C, 300 rpm, and 0.5 VVM of air. When oxygen became limiting (DO = < 20%), a feedback cascade of mixing and aeration was engaged (300–800 rpm and 0.5–1.0 VVM, respectively). The runs were performed as follows: overnight starters were used and diluted into the fermenters to OD_600_ ~ 0.1 and grown as above. At the indicated time points, OD was measured also offline after a 5” sonication using the Sonics – VibraCel sonicator with a micro-tip (at 80% pmt) to break clamps of cells. Residual glucose levels were also measured offline using Accu-Chek Sensor strips (Roche).

### Reanalysis of gene expression data

Processed data was downloaded from Gene Expression Omnibus (GEO) with accession number GSE14708 [18]. For each gene, a *t*-test was performed between the parental and hybrid gene expression, taking the three replicates together. Enrichment was tested using XL-mHG test on the ranked list of *P* values (with parameters X = 5, L = 400).

### Proteomic analysis of diploids and hybrids GFP-tagged collections

*S. cerevisiae* 4742 Matα HO::Nat^r^ and *S. paradoxus* CBS432 OS142 ﻿Matα HO::Nat^r^ were systematically mated against the GFP collection (::HIS3; the library was a kind gift from J. Weissman, University of California, San Francisco, CA [48]). Mating was performed on rich media plates, and selection for diploid cells was performed on plates with clonNAT Nourseothricin (Werner) and lacking HIS. To manipulate the collection in high-density format (384), we used a RoToR bench top colony arrayer (Singer Instruments). Automatic high-throughput microscopy screens and analyses were performed as described previously [49].

### Creating hemizygote collections

The *S. cerevisiae* MATa haploid deletion collection of non-essential genes [31, 32] consisting of 5171 open reading frames replaced with G418 resistance was systematically mated with *S. paradoxus αNat* (Matα strain of *S. paradoxus* CBS432 OS142) to produce a collection of hemizygote hybrids, and with *S. cerevisiae* 4742 to produce hemizygote *S. cerevisiae* diploids. The strains were organized in 96-well plates (Nunc) and replicated onto YPD agar plates pre-plated with the Mat α strain. The plates were incubated overnight at 30 °C to allow mating to occur and then replicated to double selection plates (YPD + 0.1 mg/mL Nat + 0.2 mg/mL G418) to select for diploid progeny. The final collection consisted of 4484 hemizygote strains.

### Pooling

The hemizygote *S. cerevisiae* and hybrid libraries were grown on YPD agar plates with antibiotic selection of G418 (200 μg/mL) for the hemizygote *S. cerevisiae* library and with G418 and Nat (200 μg/mL each) for the hybrid library. After 2 days of growth at 30 °C, the libraries were replicated in triplicate. Following 2 days of growth at 30 °C, colonies had a good and uniform size and cells were soaked off the plates by adding 10 mL of YPD liquid media + G418 (200 μg/mL) to each plate and gently scraping the cells off the plate to resuspend the cells. After the cells were resuspended, a fixed volume of 100 μL of cell suspension was taken from each plate. These samples were all mixed together, diluted to OD_600_ ~ 50 and kept at –80 °C with a final concentration of 15% glycerol. From the three copies for each library, the two best looking plates were taken and pooled in parallel; these represent the biological replicates used for each library.

### Growth experiment of hemizygote libraries

The pools were thawed on ice. Then, for recovery, they were diluted to OD_600_ = 0.1 in 30 mL of YPD and grown at 30 °C for 3 hours, completing roughly 1.5–2 doublings. Subsequently, OD_600_ was measured, and the pools were diluted to OD_600_ = 0.01 in a volume of 200 mL. At this point, the first time sample was removed, depicting the reference time point measurement. Along the experiment, samples were taken every 3–4 generations, giving on average nine time points over a total of 30 generations. Before the cells reached OD_600_ = 1, they were diluted to OD_600_ = 0.01 in order to keep them in exponential phase throughout the experiment. For sampling, 6 × 10^7^ cells were removed twice (serving as technical replicates), spun down, supernatant removed, and the pellet saved at –20 °C. Given the size of the pools, every strain should have been sampled ~10,000 times, assuming equal representation, minimizing sampling bias.

### Genomic DNA purification and PCR amplification of barcodes

Genomic DNA was purified using Epicenter MasterPureTM Yeast DNA Purification Kit. Next, 100 ng of genomic DNA was used for the PCR amplification, which was conducted in a total of 50 μL using KAPA HiFi HotStart PCR Kit with the following conditions: 95 °C/4 min; 23 cycles of 98 °C/20 s, 65 °C/15 s, 72 °C/15 s; followed by 72 °C/5 min. Barcodes were added to the general primers. For the upstream barcodes the following primers were used: (forward) 5’-NNNNNGATGTCCACGAGGTCTCT-3’ and (reverse) 5’-NNNNNGTCGACCTGCAGCGTACG-3’. For the downstream barcodes the following primers were used: (forward) 5’-NNNNNCGAGCTCGAATTCATCGAT-3’ and (reverse) 5’-NNNNNCGGTGTCGGTCTCGTAG-3’. The PCR product was then purified with Qiagen MinElute 96 UF PCR Purification Kit. Following PCR purification, DNA was quantified with the Invitrogen Quant-iT dsDNA BR Assay Kit and then equal amounts of DNA were pooled.

### Sequencing analysis and growth rate quantification

The five-base multiplexing tag allowed for post-sequencing assignment of each read to a particular measurement (time point and pool) using FASTX_Barcode_Splitter allowing zero mismatches. Using cutadapt and FASTX_trimmer all the multiplex barcodes and the common primer sequences were removed to be left with the strain barcodes only. The strain barcodes were aligned to a reference table using bowtie allowing for one mismatch. All barcode counts (B1 for the barcode upstream of the selection marker and B2 for the barcode downstream of the selection marker) were separately normalized by total coverage (read counts were divided by total read count per time point and pool, multiplied by 10^6^ to get number of transcript per million (TPM)). For all conditions except for YPD + 8% ethanol, the PCR and sequencing was performed twice as mentioned above (serving as technical replicates). These technical repeats were averaged after total read normalization by taking the average in log scale. For each strain that had more than 30 reads at the first time point, growth rates were extracted by fitting a linear model (log_2_(TPM + 1) ~ generations) using the rlm function in R (MASS package) for B1 and B2 separately. The reported growth rate per strain is the mean of B1 and B2, if both B1 and B2 growth rates were defined, otherwise just the identified growth rate (B1 or B2). Under the growth condition of YPD + ethanol, only the first five time points were taken for calculating the growth rates, as the population size remained stable over this range. For each condition, growth rates were standard normalized (mean was subtracted and divided by standard deviation). A strain was considered to have a reduced growth rate if the *Z* Score was below –1.5 in both biological replicates. Some strains could never be identified, presumably because of mutations in the barcode or the primer sequences. The number of strains that were identified in our screen in at least one condition was 4004 out of 4484 and 5362 out of 6330 for the hybrid and the *S. cerevisiae* pool, respectively. The hybrid pool is smaller to begin with because it excludes any essential strains.

### Growth assay in ethanol toxicity

Newly transformed 4741 MKS1::Hyg^r^, RTG2::Hyg^r^ and KanMX-pTDH3-RTG2 strains were mated with *S. paradoxus* Matα HO::Nat^r^ with the deletions MKS1::Hyg^r^ or RTG2::Hyg^r^ to create hybrids. After mating, they were grown on selection plates in order to select for diploid progenies. The strains were grown in YPD overnight in optimal conditions and back-diluted by adding 20 μL of the stationary culture to 5 mL YPD. After allowing the cultures to reach log phase, they were washed with YPD + 7.5% ethanol and diluted to OD_600_ = 0.05 in YPD + 7.5% ethanol media. The cultures were back-diluted to OD_600_ = 0.05 if, during an OD_600_ measurement, the culture OD_600_ was higher than 0.35.

## Additional files

Additional file 1: Figure S1. Perturbed regulated cell-cycle delay in the hybrid. (A) Distribution of the generation time of the hybrid and its diploid parents (N = 12). (B) Distributions of durations of the unbudded (G1) and budded (S + G2 + M) cell cycle phases in the hybrid and its diploid parents. Data as presented in Fig. 1c (N = 200 cells). (PDF 185 kb)

Additional file 5: Table S1. Allele-specific gene expression values for *S. cerevisiae*, *S. paradoxus*, and the hybrid in three replicates. The fold-change describes the fold-change of the expression of the hybrid over the mean of the expression of the parental strains *S. cerevisiae* and *S. paradoxus*. (XLSX 711 kb)

Additional file 6: Figure S2. Perturbed glucose-dependent respiration repression in the hybrid. (A, B) The hybrid consumes oxygen in the presence of glucose, but not its diploid parents. Shown are additional measurements for the experiments described in Fig. 2b, as indicated. (C) Hybrid mitochondria are larger and contain more cristae. Quantification of mitochondria area and cristae of the hybrid and the diploid parents, from electron microscopy images. (D) Heterosis is lost when respiration is inhibited. Growth curves for the data shown in Fig. 2e (N = 3). (PDF 215 kb)

Additional file 7: Table S2. Summary of the proteins that appeared with a change in localization between *S. cerevisiae* and hybrid. (XLSX 98 kb)

Additional file 8: Table S3. A table of all the read counts for each barcode at each time point and each pool of strains. Each experimental and technical condition is in a different tab. Upstream and Downstream represent the two barcodes for each strain (upstream and downstream of the selection marker). If a condition had technical replicates in the quantification of each strain, the tab name will specify Upstream1/Downstream1 and Upstream2/Downstream2. (XLSX 20423 kb)

Additional file 9: Table S4. Z-scores of inferred growth rates for each experimental condition, each pool and for all strains. (XLSX 1770 kb)

Additional file 10: Figure S3. Genome-wide screen for alleles contributing to hybrid growth. (A) Distribution of effects. Shown is the cumulative number of strains as a function of the indicated Z-score value. Dashed line indicates Z-score = –1.5, with the number of strains passing this threshold indicated in parenthesis for each pool. (B) Strains showing a significant effect in at least one condition. Same as Fig. 4f for the 808 genes that showed a significant effect in at least one condition or background (Z-score < –1.5 in both replicates). Genes were classified into different functional groups based on literature search (Additional file 11: Table S5). (C) Sensitivity to genes involved cell wall, protein and lipid metabolism. Same as Fig. 4f for the indicated strains. (D) Condition-dependent hoploinsufficiency. Previously identified haploinsufficient genes [34] were recovered in YPD and sorbitol but not in other growth conditions. (PDF 2466 kb)

Additional file 11: Table S5. Z-scores of inferred growth rates for each experimental condition and each pool for strains that grew slower (Z-score < –1.5 in both replicates) in at least one condition and genetic background. The categories and gene groups we assigned each strain to are provided. Categories are major functional groups, whereas gene groups are subgroups within the categories. The ‘Cell.Size.Upon.Deletion’ column states the phenotype on cell size when a gene is deleted, as determined by [50]. (XLSX 270 kb)

Additional file 12: Figure S4. Hybrid growth did not slow-down during ethanol stress. (A) For the *S. cerevisiae* pool, long-term survival in ethanol stress correlates only partially with initial rapid growth. Most strains that have high initial growth rate (defined as Z-score > 2 in both *S. cerevisiae* experiments) were survivors. However, there were many strains that were consistently highly abundant at the end of the experiment but did not show very high growth rate initially. Ribosomal genes as well as known haploinsufficient genes were enriched in the survivor pool. Two additional groups of interest were highlighted, mitochondrial genes and chromatin remodelers. (B) Hemizygote strains surviving the ethanol stress. Shown is the time-course data (as in Fig. 4c) for strains that maintained high abundance during late growth in ethanol stress. Data was normalized for time point 0, and then population median was subtracted. (C) Hybrid shows high sensitivity to retrograde signaling. Same as Fig. 4f for the indicated strains. (D) Increasing retrograde signaling improves hybrid growth under ethanol stress. Cultures were diluted periodically to maintain the cells in logarithmic growth. Shown are hemizygote hybrids deleted of their *S. cerevisiae* copy. MKS1 is a negative regulator, whereas RTG2 is an activator of retrograde pathway. (PDF 1239 kb)

Additional file 13: Table S6. Strain abundances (normalized barcode reads) for all time points 1–9 for *S. cerevisiae* pool A and *S. cerevisiae* pool B in YPD + 8. (XLSX 1573 kb)
