# Supplementary figures and images for: Heterosis as a consequence of regulatory incompatibility
Source: BMC Biol. 2017 May 11;15:38. doi: 10.1186/s12915-017-0373-7 (PMC5426048; doi:10.1186/s12915-017-0373-7)

FIGURE S1

A

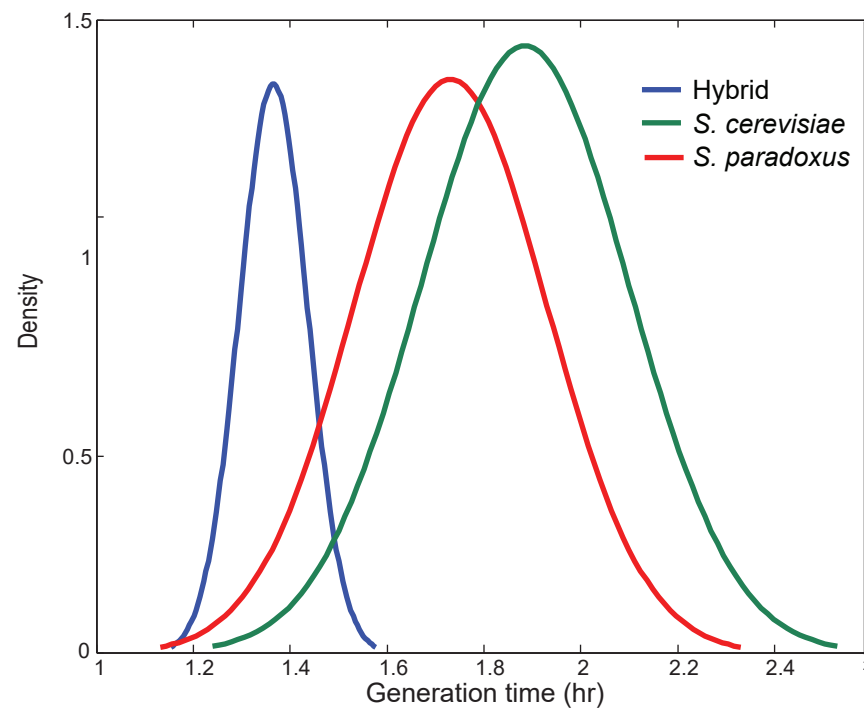

B

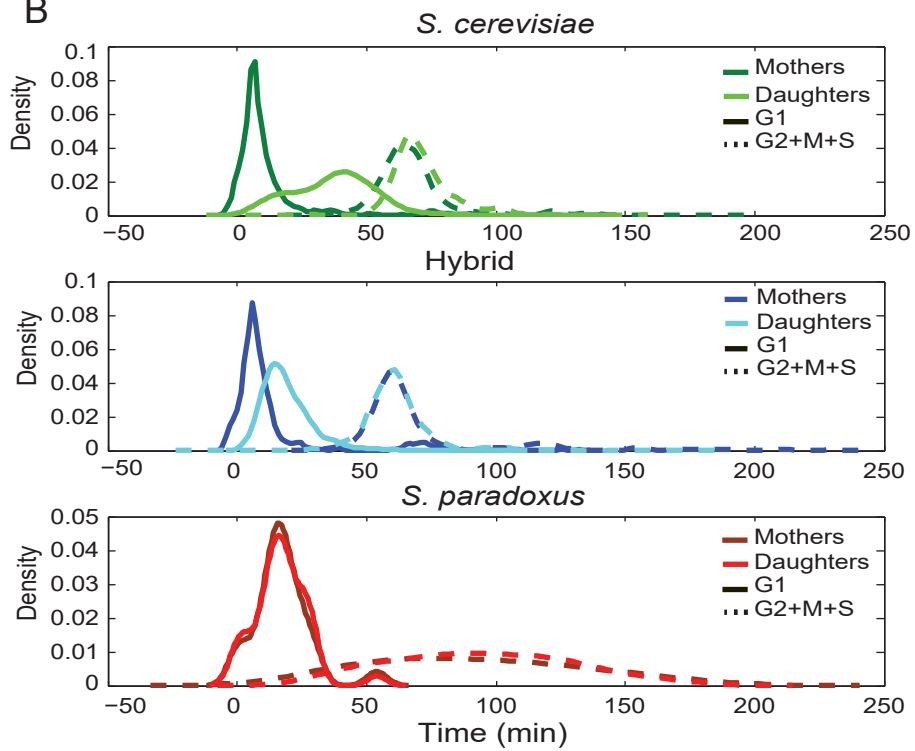

Supplement: Supplementary file 4 — Perturbed regulated cell-cycle delay in the hybrid. (A) Distribution of the generation time of the hybrid and its diploid parents (N = 12). (B) Distributions of durations of the unbudded (G1) and budded (S + G2 + M) cell cycle phases in the hybrid and its diploid parents. Data as presented in Fig. 1c (N = 200 cells). (PDF 185 kb) [file 12915_2017_373_MOESM1_ESM.pdf]

# FIGURE S2

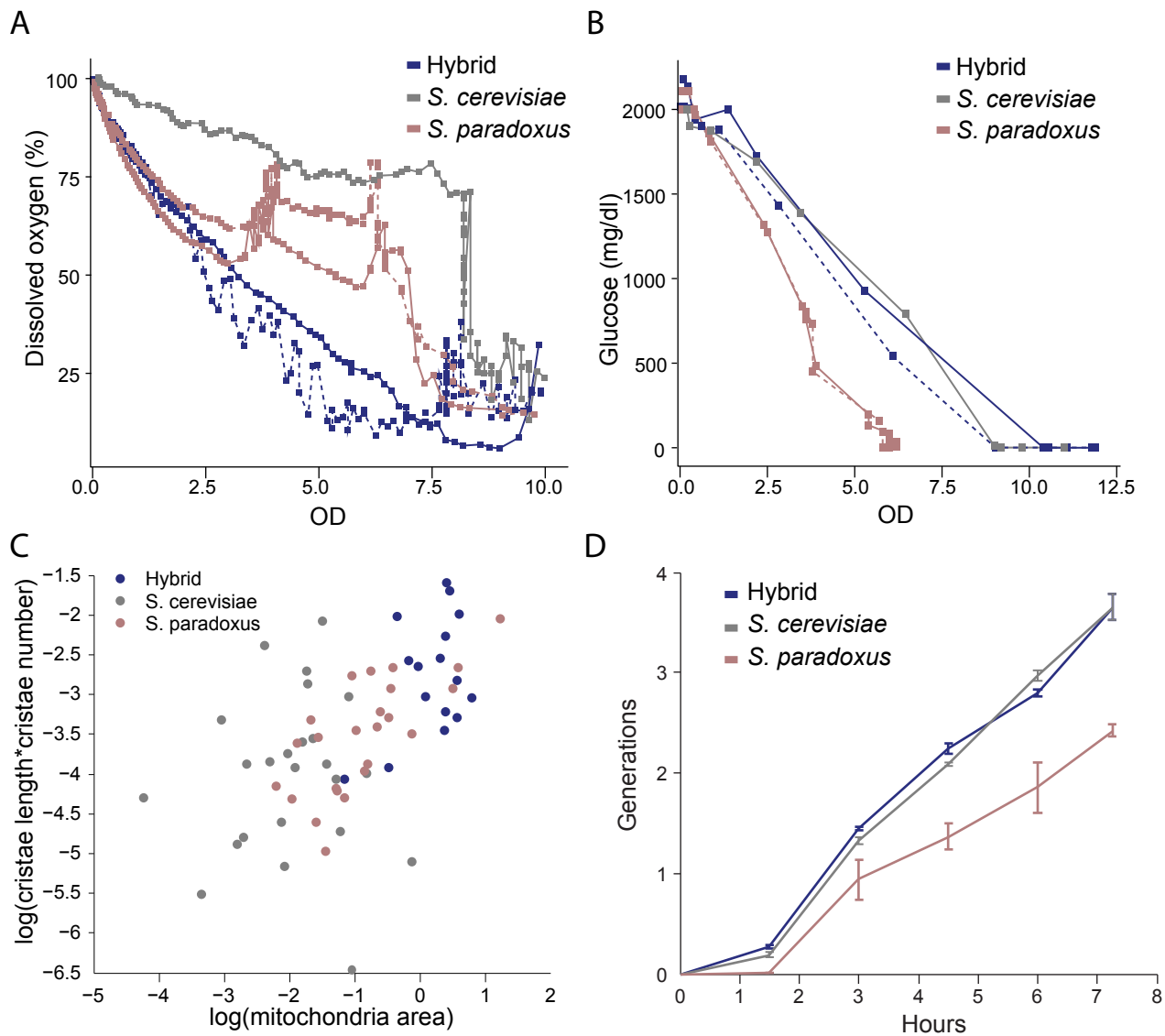

Supplement: Supplementary file 6 — Perturbed glucose-dependent respiration repression in the hybrid. (A, B) The hybrid consumes oxygen in the presence of glucose, but not its diploid parents. Shown are additional measurements for the experiments described in Fig. 2b, as indicated. (C) Hybrid mitochondria are larger and contain more cristae. Quantification of mitochondria area and cristae of the hybrid and the diploid parents, from electron microscopy images. (D) Heterosis is lost when respiration is inhibited. Growth curves for the data shown in Fig. 2e (N = 3). (PDF 215 kb) [file 12915_2017_373_MOESM6_ESM.pdf]

# FIGURE S3

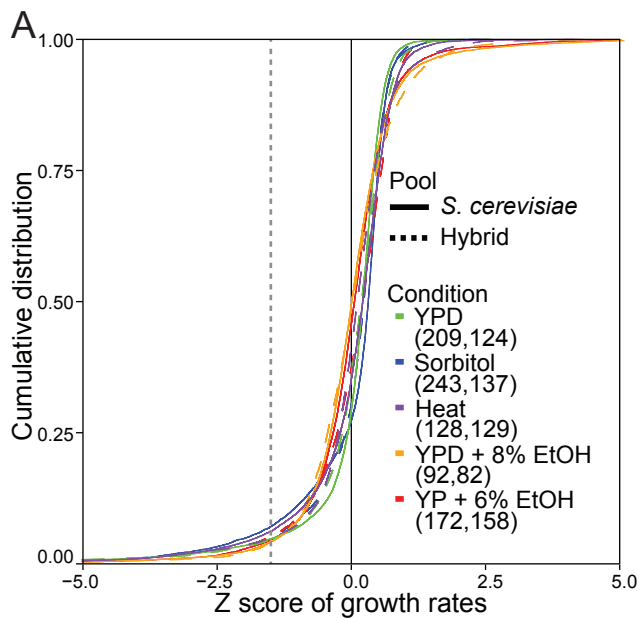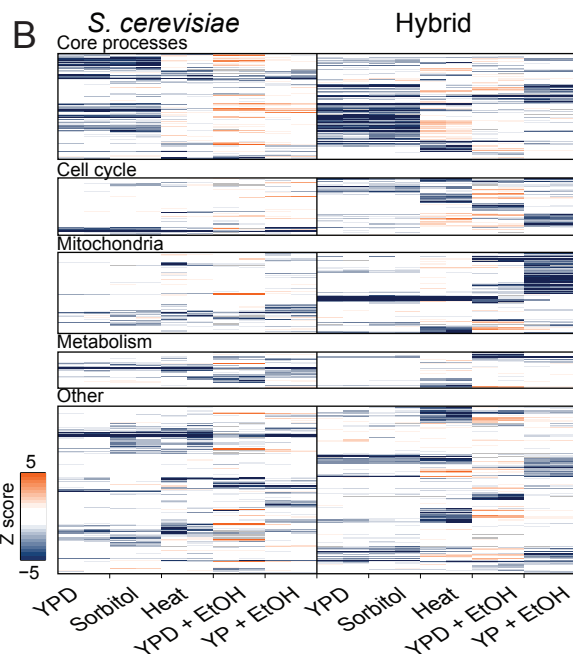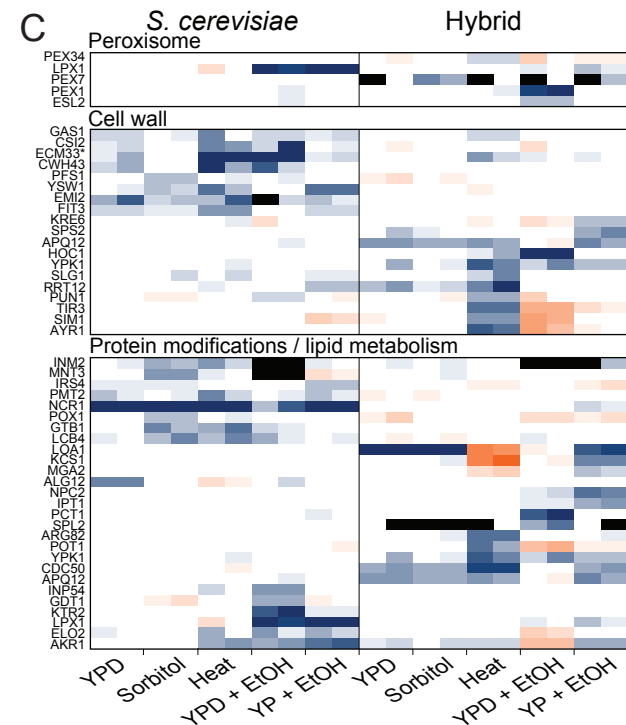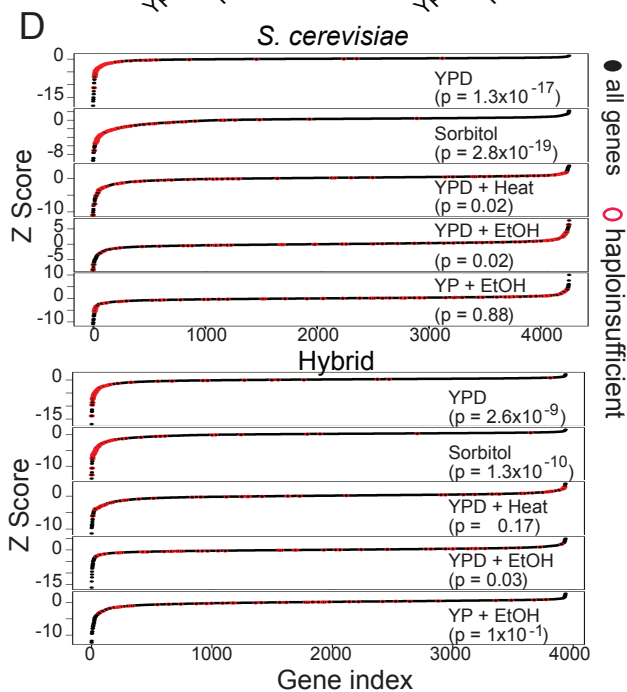

Supplement: Supplementary file 10 — Genome-wide screen for alleles contributing to hybrid growth. (A) Distribution of effects. Shown is the cumulative number of strains as a function of the indicated Z-score value. Dashed line indicates Z-score = –1.5, with the number of strains passing this threshold indicated in parenthesis for each pool. (B) Strains showing a significant effect in at least one condition. Same as Fig. 4f for the 808 genes that showed a significant effect in at least one condition or background (Z-score < –1.5 in both replicates). Genes were classified into different functional groups based on literature search (Additional file 11: Table S5). (C) Sensitivity to genes involved cell wall, protein and lipid metabolism. Same as Fig. 4f for the indicated strains. (D) Condition-dependent hoploinsufficiency. Previously identified haploinsufficient genes [34] were recovered in YPD and sorbitol but not in other growth conditions. (PDF 2466 kb) [file 12915_2017_373_MOESM10_ESM.pdf]

# FIGURE S4

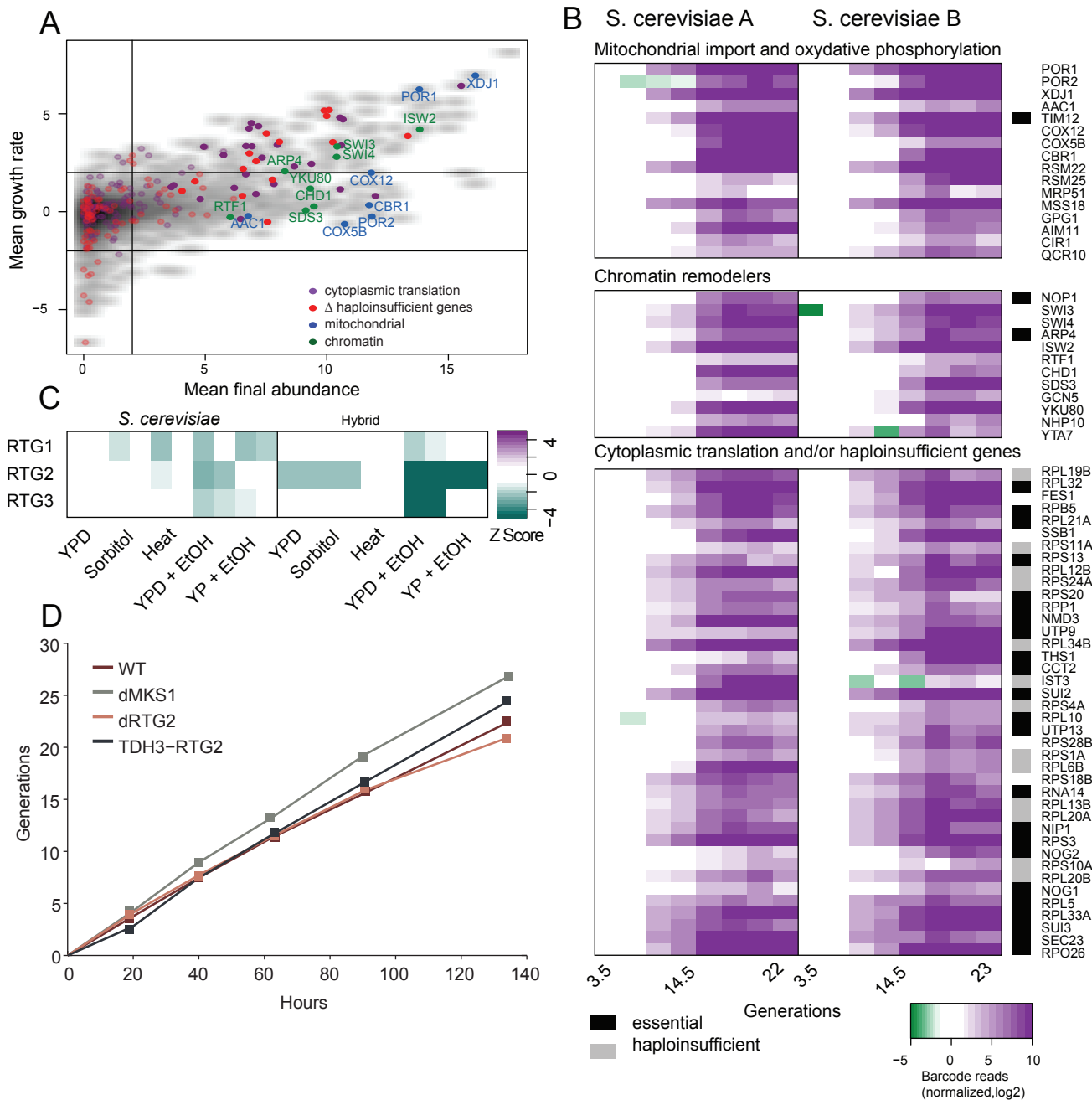

Supplement: Supplementary file 12 — Hybrid growth did not slow-down during ethanol stress. (A) For the S. cerevisiae pool, long-term survival in ethanol stress correlates only partially with initial rapid growth. Most strains that have high initial growth rate (defined as Z-score > 2 in both S. cerevisiae experiments) were survivors. However, there were many strains that were consistently highly abundant at the end of the experiment but did not show very high growth rate initially. Ribosomal genes as well as known haploinsufficient genes were enriched in the survivor pool. Two additional groups of interest were highlighted, mitochondrial genes and chromatin remodelers. (B) Hemizygote strains surviving the ethanol stress. Shown is the time-course data (as in Fig. 4c) for strains that maintained high abundance during late growth in ethanol stress. Data was normalized for time point 0, and then population median was subtracted. (C) Hybrid shows high sensitivity to retrograde signaling. Same as Fig. 4f for the indicated strains. (D) Increasing retrograde signaling improves hybrid growth under ethanol stress. Cultures were diluted periodically to maintain the cells in logarithmic growth. Shown are hemizygote hybrids deleted of their S. cerevisiae copy. MKS1 is a negative regulator, whereas RTG2 is an activator of retrograde pathway. (PDF 1239 kb) [file 12915_2017_373_MOESM12_ESM.pdf]
